# MicroRNA-34 family: a potential tumor suppressor and therapeutic candidate in cancer

**DOI:** 10.1186/s13046-019-1059-5

**Published:** 2019-02-04

**Authors:** Lu Zhang, Yi Liao, Liling Tang

**Affiliations:** 1 Key Laboratory of Biorheological Science and Technology, Ministlry of Education, College of Bioengineering, Chongqing University, Chongqing, 400044 China; 2 Department of thoracic surgery, Southwest Hospital, Army Medical University (Third Military Medical University), Chongqing, China

**Keywords:** miR-34, Dysregulation, EMT, Tumor-suppressive, Oncotherapy

## Abstract

MicroRNA-34 (miR-34) has been reported to be dysregulated in various human cancers and regarded as a tumor suppressive microRNA because of its synergistic effect with the well-known tumor suppressor p53. Along with the application of MRX34, the first tumor-targeted microRNA drug which based on miR-34a mimics, on phase I clinical trial (NCT01829971), the significance of miR-34 is increasingly recognized. miR-34 plays a crucial role on repressing tumor progression by involving in epithelial-mesenchymal transition (EMT) via EMT- transcription factors, p53 and some important signal pathways. Not only that, numerous preclinical researches revealed the giant potential of miR-34a on cancer therapy through diversiform nano-scaled delivery systems. Here, we provide an overview about the function of miR-34 in various cancers and the mechanism of miR-34 in tumor-associated EMT. Furthermore, its potential role as a microRNA therapeutic candidate is also discussed. Notwithstanding some obstacles existed, the extensive application prospect of miR-34 on oncotherapy cannot be neglected.

## Background

MicroRNAs (miRNAs or miRs) are a class of high-conserved, small (about 22 nucleotides in length), single-stranded noncoding RNAs. They can bind with 3′- untranslated regions (UTRs) of messenger RNAs (mRNAs) to inhibit mRNA translation or induce mRNA degradation, thus silencing gene expression at the post-transcription level. A single miRNA may regulate hundreds of target mRNAs which possess same short recognition region, simultaneously, the 3’-UTR of most mRNAs exist more than one binding site for different miRNAs. Since Lee et al. [1] discovered the first miRNA *lin-4* in 1993, the researches on miRNAs have been greatly progressed, and the function of miRNAs also gradually becomes clear. miRNAs have been reported to control the expression of approximately 30% human essential genes which are mostly essential for normal survival and development [2]. Therefore, by regulating these fundamental target genes, miRNAs can involve in various kind of signal pathways to modulate great quantity of important biological processes, such as cell proliferation [3], metastasis [4], apoptosis [5], senescence [6], differentiation [7], autophagy [8] and immune response [9]. Moreover, miRNAs have been found dysregulation under lots of pathological conditions, such as neurodegenerative diseases [10], cardiovascular diseases [11] and leukemia [12], especially in cancer [13]. The functions of miRNAs depend on what pathological type and physiological environment they are in, may as tumor suppressors to inhibit tumor cell proliferation, or as oncogenes to induce tumorigenesis.

As a member of microRNA, miR-34 has been detected to be dysregulated in various cancers, and also is the first miRNA that demonstrated to be directly regulated by the tumor suppressor p53 [14]. Thus miR-34 family is known to inhibit tumorigenesis. The expression of miR-34 family relies on endogenous expression or mimics transfection. Plenty of mature miR-34 has been observed inactive in several kind of cancer cells because of the lack of a 5′-phosphate. However, when given a DNA-damaging stimulus to these cells, inactive miR-34 can be rapidly activated through 5′-end phosphorylation [15]. The dysregulation of miR-34 in a variety of cancers makes it be the focus of concern. Furthermore, a large quantity of experimental data showed that miR-34 could influence EMT. One study presented that increasing expression of miR-34a by transfecting miR-34a mimics could inhibit the invasion ability of bladder cancer cell 5637-M [16]. Another study showed that inducing expression of pri-miR-34a by doxycycline could result in the down-regulation of vimentin and the up-regulation of E-Cadherin in human colon cancer cell SW480 [17]. These results suggested that miR-34 family members can regulate EMT negatively to inhibit proliferation and invasion in tumor cells.

EMT is a common cellular biological process. In this process, epithelial cells lose their morphologies and adhesion ability and obtain a mesenchymal phenotype. EMT can be described into three categories according to physiological tissue contexts, and the most well-defined type is the EMT in cancer progression [18]. Primary tumor cells can acquire migration and invasive abilities through EMT and form metastases. EMT is an important process in tumor evolution undoubtedly, it provides the possibility for tumor cells to adapt tumor microenvironment. The activation conditions of EMT are diverse. Appropriate cellular environments, cytokines and extracellular signals all may induce EMT. In addition, EMT-associated transcription factors (EMT-TFs) are also essential for the activation of EMT. There are three most promising positive EMT-TFs, zinc-finger transcription factors SNAIL family (SNAIL1, SNAIL2 and SNAIL3), ZEB transcription factors (ZEB1 and ZEB2) and basic helix-loop-helix (bHLH) transcription factors TWIST family (TWIST1 and TWIST2) [19]. Increasing number of studies indicated that microRNAs can combine with EMT-TFs to form double-negative feedback loop, thus interfering EMT [20]. It is reported that SNAIL 3’-UTR owns a conserved sequence which could match with miR-34 [18]. Besides, miR-34 also can control EMT via other approaches. In short, miR-34 is a vital negative regulator for EMT in cancer.

In this review, we focus on the function of miR-34 in various cancers and the underlying mechanism. More importantly, the broad application prospect of miR-34a as a promising therapeutic candidate is also discussed.

### The function of miR-34 in cancer

miR-34 family has three members, including miR-34a, miR-34b and miR-34c. Interestingly, these three miR-34 family members are encoded by two different transcriptional units. miR-34a is located at chromosome 1p36.22 and has an unique transcript, while miR-34b and miR-34c hold one transcript in common which located at chromosome 11q23.1 [21]. Compare the sequence of these three members and find that miR-34a shows high identity with miR-34b and miR-34c. The seed region which between second to ninth nucleotide at the 5′-end of mature miRNAs is the guarantee for recognizing mRNA 3’-UTR. Interestingly, the seed sequence of miR-34a and miR-34c is identical, indicating that they hold similar mRNA target, but miR-34b is a little different [22]. Except in lungs, the expression of miR-34a is higher than miR-34b/c in most human tissues. miR-34a shows highest expression level in brain, while miR-34b/c mainly in lung [23]. Whereas, in various cancers, miR-34a and miR-34b/c expression level is much lower because of the CpG methylation [24]. Notably, miR-34 is the well-known miRNA which regulated by tumor suppressor p53. And it is known as a kind of tumor suppressive miRNA because of the synergistic effect with p53 in antitumor and the low expression level in various cancers.

### miR-34 in colorectal cancer

Many studies have indicated that the expression level of miR-34 family in colorectal cancer tissues was lower than adjacent non-tumor tissues [25, 26]. Roy et al. [27] found that compared with normal tissues, miR-34a and miR-34c were down-regulated in human colon cancer tissue, and the reason for down-regulating was promoter hypermethylation. Notwithstanding the cause of decrease expression of miR-34 is hypermethylation, but not only that, SUMOylation has also been verified to regulate miR-34b/c level in colon cancer [28]. The dysregulation of miR-34 suggests its potential role as biomarker. In the Apc^Min/+^ mice model which deleted miR-34a or miR-34b/c, the number of tumors and risk of death were shown to be significantly increased [29]. Moreover, miR-34a or miR-34b could inhibit cell migration and invasion in colorectal cancer (CRC) cells [30]. These findings demonstrated the tumor-suppressive function of miR-34 in CRC. However, far from the down-regulation of miR-34 in CRC, several researches presented exactly opposite data and perspectives. Two independent studies analyzed large quantity of colon cancer patients tissue samples, miR-34 family three members were all observed up-regulated in colon tumors compared with the corresponding normal tissues, and the high expression of miR-34 was correlated with poor prognosis [31, 32]. The conflict results come from the different tumor microenvironment. The up-regulation of miR-34 may occur in cancer tissues with inflammation [31].

### miR-34 in prostate cancer

As the leading malignant tumor diagnosed among men, prostate cancer (PCa) has always been receiving a lot of attention. It is reported that miR-34 is down-regulation in human prostate cancers compared with corresponding benign tissues and plays the negative role in prostate cancer essential metabolic process. Liang et al. [33] showed the decrease of miR-34a in 20 human primary prostate cancer specimens, meanwhile they found that miR-34a can regulate Wnt signal pathway negatively to inhibit EMT-associated migration and invasion. Furthermore, compared with PC-3 (a high metastatic potential PCa cell line), miR-34a and miR-34b/c expression levels were increased in DU-145 (a moderate metastatic potential PCa cell line), while the uptrend of miR-34b and miR-34c was markedly higher than miR-34a [34]. Moreover, the overexpression of miR-34b or miR-34c in PCa cells revealed pronounced inhibition in cell migration, invasion and proliferation, whereas showed no influence on apoptosis [34]. It uncovered the crucial effect of miR-34 on PCa metastasis.

### miR-34 in breast cancer

In breast cancer (BC), the most common cancer among women, the expression levels of miR-34 family members were all detected down-regulated compared with healthy tissues [35, 36]. Zeng et al. [37] explored the expression of miR-34 three members in 173 triple-negative breast cancer (TNBC) patients, and found that the patients with low expression of miR-34a and miR-34c showed worse overall survival. Moreover, miR-34a and miR-34c were associated with the metastasis of BC. Compared with the non-metastatic BC cells, the expression of miR-34a and miR-34c was much lower in metastatic BC cells. And in vitro experiments showed that the overexpression of miR-34a or miR-34c repressed the migration and invasion of BC cells [38]. miR-34c is the well-studied member of miR-34 family in BC. As a kind of tumor suppressor, miR-34c exhibits the crucial role in inhibiting cellular self-renewal [39], repressing cell proliferation [36] and inducing G2/M cell cycle arrest [40]. In addition, miR-34a reduced BC stem cell properties and chemoresistance. Not only that, BC-bearing nude mice which treated with miR-34a showed significant inhibition of tumor formation [41]. Although miR-34b holds similar function with miR-34c because of the common transcript, they exert slight differences in several biological functions. For example, miR-34b showed a minor effect on cell growth, apoptosis and migration than miR-34c in breast cancer cell line MDA-MB-231 [42].

### miR-34 in lung cancer

The expression of miR-34 in lung cancer has also been analyzed in an abundant of studies. In non-small cell lung cancer (NSCLC) including squamous cell carcinoma (SCC) and lung adenocarcinoma (LAC), the expression of miR-34 family three members were all decreased compared to normal tissues/cells [43–45]. By investigating the expression of miR-34 members in plasma and tumor tissue of 196 NSCLC patients, something interesting was emerged. The high expression of miR-34a and miR-34c in both plasma and tumor tissues was associated with prolonged overall survival and disease-free survival compared with low expression [46]. It showed the possibility of regarding miR-34a and miR-34c as potential prognostic markers in NSCLC. Moreover, metastatic LAC exhibited lower expression level of miR-34b/c than non-metastatic LAC, indicating that miR-34b/c can suppress the metastasis ability in LAC cells, while there was no obvious difference in miR-34a [47]. However, in LAC tumor tissues, miR-34a was detected decreased. At the same time, the expression of miR-34b/c was too low to detect [48]. Besides, in small cell lung cancer (SCLC), miR-34a and miR-34b/c were down-regulated because of the methylation. More importantly, miR-34b/c exhibited higher frequency of methylation compared with miR-34a [49, 50]. These results may demonstrate that miR-34b/c performs its functions primarily in lung tissues.

### miR-34 in liver cancer

Many experimental studies have reported that miR-34 was dysregulated in liver cancers. Jiao et al. [51] detected the expression of miR-34 in 78 children hepatoblastoma (HB), found that three miR-34 family members were all significantly up-regulated in tumor tissues compared with non-tumor tissues. The same uptrend of miR-34 was also showed in hepatocellular carcinoma (HCC) [52]. However, not all experimental results are always completely consistent. Compared with adjacent healthy tissues, miR-34a and miR-34b were demonstrated to be lower in 30 of HCC tumor tissues, simultaneously, the methylation level of miR-34 family members in tumor tissues was detected to be higher than corresponding noncancerous tissues, indicating the reason for silencing miR-34 in HCC is still the promoter methylation [53].

### miR-34 in osteosarcoma

Osteosarcoma (OSA) mostly occurs in children and adolescents under 20 years of age. The mRNA expression level detection, flow cytometry and immunohistochemistry staining results have demonstrated that miR-34 family members were all down-regulated in OSA compared with healthy bone tissues, and the low expression of miR-34a was an independent marker for poorer disease-free survival in OSA patients [54]. Moreover, miR-34a can promote apoptosis and cell cycle arrest at G0/G1 phase by binding with DUSP1 in OSA, indicating that miR-34a may as a novel tumor suppressor in OSA pathogenesis [55]. Additionally, miR-34b also shows the anti-tumor effect in OSA. Mice which suffered with OSA exhibited smaller tumor volume and more apoptotic cells after treating with miR-34b, suggesting that miR-34b could inhibit growth and induce apoptosis of OSA [56].

Besides above described cancer types, miR-34 family members have been reported dysregulated in other cancers. The expression of miR-34 in various solid tumors is listed in Table 1.

**Table 1:** miR-34 expression in human solid tumors

Tumor type	miR-34 family member	Number of tumor samples	Expression level compared with normal tissues	Ref.
BC	miR-34 family	173	Down	[37]
BC	miR-34a	134	Down	[94]
ccRCC	miR-34a	45	Up	[107]
ccRCC	miR-34a, miR-34b	17	Up	[108]
Colon Cancer	miR-34a, miR-34c	10	Down	[27]
Colon Cancer	miR-34a	40	Down	[109]
Colon Cancer	miR-34 family	272	Up	[31]
CRC	miR-34a	100	Down	[25]
CRC	miR-34b/c	17	Down	[110]
CRC	miR-34a	109	Up	[32]
EHCC	miR-34a	27	Down	[81]
EOC	miR-34 family	83	Down	[111]
ESCC	miR-34a	16	Down	[112]
ESCC	miR-34b	88	Up	[113]
Gastric Cancer	miR-34a	164	Up	[114]
Gastric Cancer	miR-34a	20	Up	[115]
Gastric Cancer	miR-34a	39	Down	[116]
Gastric Cancer	miR-34b	72	Down	[117]
GBC	miR-34a	77	Down	[118]
Glioma	miR-34a	21	Down	[119]
Glioma	miR-34c	18	Down	[120]
HB	miR-34 family	78	Down	[51]
HCC	miR-34a, miR-34b	30	Down	[53]
HCC	miR-34 family	57	Up	[52]
HNSCC	miR-34a	15	Down	[121]
LSCC	miR-34a	69	Down	[122]
MTC	miR-34a	30	Up	[123]
NSCLC	miR-34a	30	Down	[43]
NSCLC	miR-34b	52	Down	[49]
NSCLC	miR-34a, miR-34c	33	Down	[45]
OC	miR-34a	133	Down	[71]
OSCC	miR-34a	35	Down	[124]
OSCC	miR-34b/c	15	Up	[125]
OSA	miR-34 family	80	Down	[54]
OSA	miR-34	34	Down	[56]
PCa	miR-34a	30	Down	[33]
PCa	miR-34 family	49	Down	[126]
PDAC	miR-34a	159	Down	[127]
PDAC	miR-34a	48	Up	[128]
Rectum Cancer	miR-34a	109	Up	[32]
SCLC	miR-34b	11	Down	[49]
SCLC	miR-34 family	6	Down	[50]
SGT	miR-34a	48	Up	[129]
UBC	miR-34a	30	Down	[130]

*ccRCC* clear cell renal cell carcinoma, *EHCC* extrahepatic cholangiocarcinoma, *EOC* epithelial ovarian cancer, *ESCC* esophageal squamous cell carcinoma, *GBC* gallbladder cancer, *HNSCC* head and neck squamous cell carcinoma, *LSCC* laryngeal squamous cell carcinoma, *MTC* medullary thyroid carcinoma, *OC* ovarian cancer, *OCSS* oral squamous cell carcinoma, *OSA* osteosarcoma, *PDAC* pancreatic ductal adenocarcinoma, *SGT* salivary gland tumor, *UBC* urothelial bladder cancer

### miR-34 in haematological neoplasm

Except various solid tumors, miR-34 family members have also been detected dysregulation in some haematological neoplasms. For example, in human acute myeloid leukemia (AML) cell lines HL-60 and THP-1, the expression of miR-34a was much lower than human normal stromal cells HS-5. And the decrease expression of miR-34a inhibited autophagy and induced apoptosis [57]. Same as miR-34a, the downtrend expression of miR-34c in AML has also been demonstrated. Compared with normal hematopoietic stem cells, miR-34c was remarkably down-regulated in AML stem cells, and miR-34c low expression was associated with unfavourable prognosis and poor therapeutic response to AML patients [12]. In addition, about 18% chronic lymphocytic leukemia (CLL) patients are deficiency of the long arm of chromosome 11 where miR-34b and miR-34c located, thus the expression of miR-34b/c is much lower in CLL [58]. Not only that, p53 has been found to be lost or mutated in a large proportion of fludarabine refractory CLL cases, and as the direct downstream target of p53, miR-34a indeed shows a low expression in CLL. Nevertheless, without the condition of p53 defection, miR-34a low expression still in connection with fludarabine refractory [59]. Notably, decreased miR-34a expression was associated with p53 aberration not only, also DNA damage response disorder and apoptosis resistance [60]. Besides in leukemia, the dysregulation of miR-34 also has been found in multiple myeloma (MM). A wide majority of MM cell lines hold miR-34b/c promoter methylation [61] and SUMOylation [28], caused transcriptional obstacle and finally resulted in miR-34b/c low expression. Moreover, miR-34a not just plays the antitumor effect directly, also has been demonstrated to enhance the anticancer effect of three anticancer agents, γ-secretase inhibitor, sirtinol and zoledronic acid, in multiple myeloma [62].

### The underlying mechanism of miR-34 in cancer metastasis

**Fig. 1:**
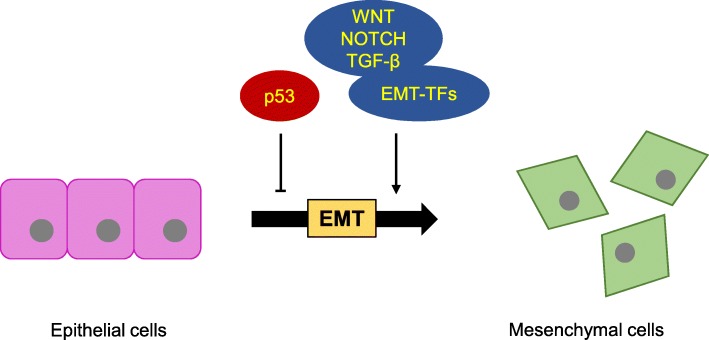
The activation condition of EMT. Epithelial cells lose their adhesion ability and obtain a mesenchymal phenotype during EMT. Tumor suppressor p53 can inhibit the transition from epithelial cells to mesenchymal cells. However EMT-associated transcription factors and some EMT-associated signal pathways are essential for EMT occurrence

Nowadays the cancer therapy is still very difficult. The difficulty lies in how to solve the problem that tumor cells spread from situ tissues to other healthy tissues. The spread of malignant tumor cells is life-threatening, therefore the underlying mechanism of tumor cells metastasis is worthy of attention. Many researches demonstrated that most tumor cells can obtain metastasis and invasion ability through EMT, resulting in a poor prognosis and even death. EMT is characterized by loss of cell polarity and a decrease expression of some epithelial markers, such as E-cadherin, cytokeratins and α-catenin, also accompanied by acquisition of cell migration and invasion ability, as well as an increase expression of some mesenchymal markers, such as N-cadherin, vimentin, fibronectin and enzyme matrix metalloproteinase family. Increasing number of findings have illustrated the negative regulation of miR-34 family members in tumor cell metastasis and invasion [63], indicating the relative relationship between miR-34 family and EMT. miR-34 family can modulate EMT by binding with pivotal target genes. As an example, miR-34c can bind with 3’UTR of Notch4 in breast tumor-initiating cells, thus inhibiting cell migration ability and the expression of vimentin and fibronectin, and promoting the expression of E-cadherin [39]. Generally speaking, there are three approaches that miR-34 negatively controls EMT to play its tumor-suppressive role (Fig. 1).

Firstly, miR-34 binds with the 3’-UTR of EMT-TFs to regulate EMT. EMT-TFs are necessary for the activation of EMT. Among these EMT-TFs, SNAIL1 is especially unique, because it can combine with the E-box sequences of E-cadherin promoter to suppress the expression of E-cadherin, thus leading to the enhancement of EMT [64]. Although SNAIL2 is unable to interact with E-cadherin directly, it inhibits E-cadherin by recruiting PRC2 and HDAC6 to the promoter of E-cadherin [65]. Furthermore, SNAIL can promote the expression of mesenchymal genes, like vimentin [66] and matrix degradation enzyme matrix metalloproteinase 9 (MMP9) [67]. Apart from regulating the expression of epithelial and mesenchymal related genes, SNAIL also plays a positive effect on other EMT-TFs [67]. The dual-reporter assay result has demonstrated that SNAIL is the direct target of miR-34 family. There is a conserved miR-34 seed-matching sequence in SNAIL1 3’-UTR. The activity of SNAIL can be modulated by miR-34, but the function of miR-34 family members also can be suppressed by SNAIL. Since SNAIL1 is the transcription factor which produce at the start of EMT, the SNAIL1/miR-34 feedback loop controls the initiation of EMT [68]. SNAIL1 is not the only EMT-TF which owns a matched sequence with miR-34 family, ZEB2 3’-UTR also exists a conserved sequence which can match with miR-34a [17]. Although SNAIL2 and ZEB1 lack the matched sequence of miR-34 family, there are still studies shown that miR-34 can indirectly down-regulate their expression [17, 69]. These above studies clearly state the inhibition of miR-34 family member on EMT-associated transcription factors, and result in the attenuation of EMT.

Secondly, miR-34a has been shown to induce p53 activation via targeting TP53 and MDM4 directly [70], but the other way round, p53 also can modulate the expression of miR-34a. Compared with p53 wild type cells, miR-34a was down-regulated in p53-mutated ovarian cancer cells [71]. In addition, when treated cells with Nutlin-3a, a kind of chemical activator of p53, the expression of miR-34a was dramatically increased [72]. These researches have showed that miR-34a expression followed the change with p53, and verified the demonstration that miR-34a is the downstream target of p53. However, miR-34b/c showed little effect on p53 activity [70]. More importantly, p53 has also been reported to diminish EMT progress by moderating the expression and activity of SNAIL1 through strengthening the expression of miR-34a [73]. In general, miR-34a, p53 and EMT can develop into an intricate network together to influence the function of each other.

**Fig. 2:**
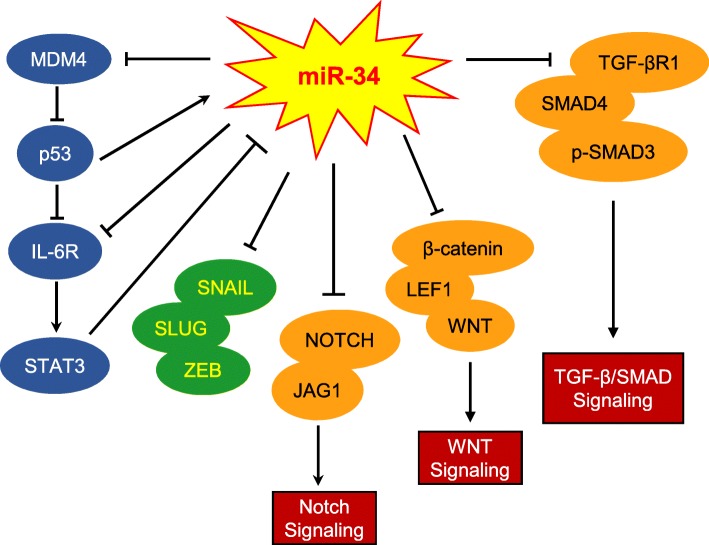
Schematic of the mechanism of miR-34 in EMT. There are two feedback loops. One is the miR-34a-p53 loop, they reinforce each other to regulate EMT. Another loop consists of miR-34a, IL-6R and STAT3, the decrease of miR-34a in cancer can induce the expression of IL-6R which increases the activity of STAT3, meanwhile STAT3 further represses miR-34a expression. Among this process, IL-6R is essential for EMT. Interestingly, p53 connects this two important loops. In addition, miR-34a regulates EMT via some vital EMT-EFs, such as SNAIL, ZEB and SLUG. Furthermore, some EMT-associated signal pathways are also the agencies between miR-34a and EMT. For example, miR-34a inhibits the expression of NOTCH and JAG1 to regulate the NOTCH pathway, the WNT pathway also is represses by miR-34a via decreasing the expression of β-catenin, LEF1 and WNT, miR-34a also can reduces the activity of TGF-β/SMAD pathway by suppressing the expression of TGF-βR1, SMAD4 and p-SMAD3. Through these essential signal pathways, miR-34a achieves the modulation of EMT

Finally, members of the miR-34 family regulate EMT can not only via EMT-TFs and tumor suppressor p53, but also via some fundamental signal pathways, such as Wnt [74], transforming growth factor beta/Smad (TGF-β/Smad) [75] and Notch [76, 77]. miR-34a could control Wnt transcriptional activity negatively by regulating multiple pathway-associated genes [78, 79]. As an important transcription factor in Wnt signal pathway, lymphoid enhancer factor-1 (LEF-1) was reported to be associated with cellular proliferation and invasion. It is reported that LEF-1 expression was decreased by miR-34a via directly binding with the 3’-UTR of LEF-1, resulted in the inhibition of migration and invasion of PCa cells and the attenuation of EMT [33]. Notably, miR-34a also indirectly suppressed LEF-1 expression through regulating β-catenin, thereby inhibiting the invasion of colon cancer cells [80]. In addition, miR-34 family members also involve in TGF-β/Smad pathway to regulate EMT. It is reported that miR-34a could inhibit the migration and invasion of cholangiocarcinoma cells by suppressing the activity of TGF-β/Smad4 pathway [81]. And miR-34b was shown to down-regulate the expression of some key genes in TGF-β pathway, for instance, TGF-β receptor 1 (TGF-βR1), p53 and phosphorylation of mothers against decapentaplegic 3 (p-SMAD3), thus weakening the migration and invasion ability of PCa cells [34]. It is well-known that activated Notch signal pathway participates in various cellular processes and strengthens the formation of several kind of neoplasms. In vivo results showed that miR-34a can bind with 3’-UTR of Notch1 and Jagged1, thus inhibiting the migration and invasion of CRC cells and decreasing the expression of mesenchymal markers [26]. Furthermore, the feedback loop which consist of miR-34a, interleukin-6 receptor (IL-6R) and signal transducer and activator of transcription 3 (STAT3) receives much attention because of the vital regulation for EMT. IL-6R mediated the activation of STAT3, an oncogenic transcription factor. Meanwhile, STAT3 could repress the expression of miR-34a via a conserved binding site which located at the first intron of miR-34a, while the inhibition of miR-34a was essential for IL-6-induced EMT [82] (Fig. 2).

### miR-34a is promising for microRNA therapeutics

Due to the dysregulation in cancers, miRNAs are classified into two types. One is tumour suppressive miRNAs, the other is oncomiRs which act as oncogenes. According to two distinct functions of miRNAs in cancer, an innovative therapeutics rely on miRNAs emerged. This novel therapeutic approach via miRNA mimics or antimiRs to modulate miRNA expression and activity in vivo [83]. As the well-studied tumor suppressor, miR-34a absolutely is an appropriate candidate for cancer therapy.

### The strategies for miR-34a systemic delivery

**Fig. 3:**
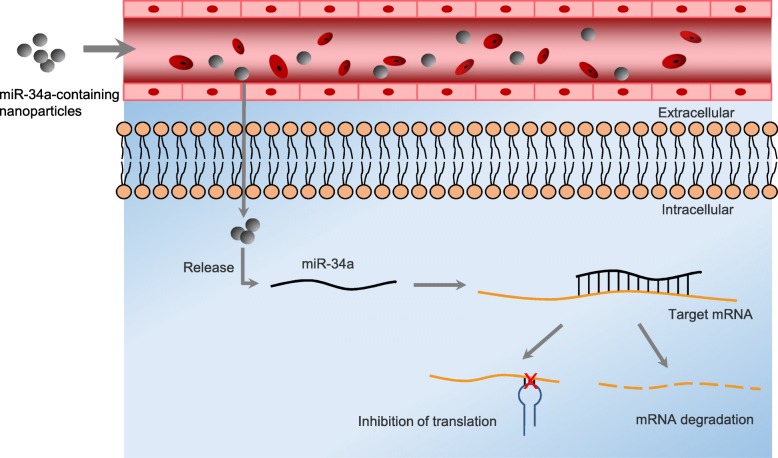
The systemic delivery of miR-34a. Nanoparticles which encapsulating with miR-34a are delivered into blood vessel intravenously. These nanocarriers overcome barriers from organs, tissues and cells to reach at target cells and release miR-34a mimics under specific intracellular environment. Then miR-34a binds with 3’-UTR of target mRNA, resulting in the degradation of mRNA or the inhibition of translation

To some extent, miRNA therapeutics is a kind of precision medicine, it can accurate to specific site to control gene expression. However, the biggest problem is the deficiency of efficient miRNAs delivery system. It is well-known that RNA is easy degraded by RNase, and RNase is abundant in serum and endocytic compartment of cells, moreover, the half-life of miRNAs is extremely short in plasma [84]. Therefore, it is hard to ensure the therapeutic efficiency when deliver miRNAs mimics or antimiRs to target cells. Up till now, there are two solutions to this problem, chemically modify nucleotides to increase miRNAs stability or apply nanocarrier delivery vehicles to avoid miRNAs degradation. However, the low membrane penetrability of chemically modified miRNAs limited the application in vivo [84]. A considerable number of in vitro studies have demonstrated the anti-tumor effect of miR-34a. Nevertheless, the application of miR-34a on clinical is restricted by inefficient target delivery. Some targeting nano-vectors are designed in order to realize the effective systemic delivery of miR-34a (Fig. 3).

Among diverse nano-scaled carriers, viral carrier is the most common and original one. The widely used viral vectors including lentivirus, retrovirus and adenovirus. Target genes can be encapsulated into virus and then transferred to the genome of target cells along with virus infection. More importantly, infection hosts generally show low immune response against viral vectors, especially lentiviral vector [85]. Therefore, as we can see, stability, perdurability and security are the major strengths of viral vectors. The miR-34a delivery system which depended on viral vectors has been applied in several diseases. For instance, lentiviral miR-34a expression system significantly increased the expression of miR-34a and induced apoptosis in MM cells. In addition, the MM xenografts formation and average size were dramatically reduced by lentiviral miR-34a in the severe combined immunodeficien (SCID) mice [86]. Lentiviral vector was also used to systemically deliver miR-34a to PCa, and results showed that the miR-34a lentiviral delivery system inhibited tumor cell metastasis and prolonged animal survival [87]. Besides, oncolytic adenovirus is also an extraordinary vehicle on account of the specific replication at tumor cells via modifying [88]. AdCN205 is an oncolytic adenovirus which modified by CR2 region deletion and replacement with human telomerase reverse transcriptase (hTERT) promoter to E1A promoter. miR-34a and tumor suppressor gene IL-24 were co-delivered via AdCN205 to HCC cells, and the infected HCC cells showed proliferation inhibition. Impressively, AdCN205-IL-24-miR-34a prominently inhibited tumor growth and induced tumor regression without tumor recurrence at HCC mice [89].

Lipid-based vector is a non-viral vector which frequently used in nucleic acid transfection. The liposome contains a hydrophilic head and a hydrophobic tail which usually combined together to influence the stability of liposome. Liposomes are popular delivery agents due to the high transfection efficiency. However, the poor stability in serum and the high toxicity restricted the application of cationic liposomes in vivo [90]. For solve these problems, Di Martino et al. [91] synthesized stable nucleic acid lipid particles (SNALPs) which held high stability and prolonged blood circulation by using of disteroylphosphatidylcholine (DSPC), cholesterol (CHOL), poly ethylene glycol 2000 (PEG-2000) and 1,2-dioleyl-3-dimethylammonium propane (DODAP). Then miR-34a was encapsulated into SNALPs to form the desired delivery system. The delivery and therapeutic efficiency of SNALPs-miR34a were tested in MM, and the results were exciting. SNALPs encapsulating miR-34a induced the expression of miR-34a and inhibited the MM xenograft growth. But not only that, SNALPs miR-34a exhibited low toxicity [91]. In order to achieve better antitumor activity, SNALPs-miR-34a system was upgraded by means of conjugating SNALPs with transferrin (Tf) and modifying miR-34a by 2’-O-methylated (OMet). Indeed, the Tf-SNALPs encapsulating OMet miR-34a prolong MM mice survival compared with previous unmodified SNALPs miR-34a delivery system [92]. Besides, the miR-34a and let-7b compound was co-delivered to (Kras^LSL-G12D/+^;p53^flx/flx^) NSCLC mice by a neutral lipid emulsion (NLE) vehicle, found that the tumor burden was significant declension [93]. Lin et al. [94] constructed a TV-miR-34a plasmid consisted of hTERT promoter-driven VP16-GAL4-WPRE integrated systemic amplifier (VISA) and miR-34a, and delivered TV-miR-34a to breast cancer stem cells (BCSC) by synthesized DODAP and CHOL liposomes. The TV-miR-34a system induced high expression of miR-34a and attenuated tumor-initiating properties in BCSC. Moreover, the BCSC-bearing tumors mice which treated with TV-miR-34a showed pronounced inhibition of tumor growth [94]. The oncotherapy of miR-34a which depended on lipid-based vectors has also been verified in other cancers, such as neuroblastoma [95] and pancreatic cancer [96].

Polymeric vector is a kind of nanocarrier which received much attention because of the low immunogenicity and cytotoxicity, ingredients variability and structural stability [97]. Some studies have reported the modulation of miR-34a in the response of tumor cell to chemotherapy [98]. In view of this, one research elucidated an innovative nanoplatform which response for acid microenvironment and high glutathione (GSH) in tumor cells to accelerate drug release so as to combat with chemoresistance. The authors conjugated polycarbonate backbone with rubone (RUB), an activator of miR-34a, and diisopropylamino ethanol to made P-RUB which could assemble into micelles by itself. And docetaxel (DTX) was encapsulated in P-RUB micellar core to form DTX/P-RUB micelles. This system could diffuse and disassemble to release DTX and RUB fleetly in condition of protonation and GSH induced disulfide bond cleavage, thus playing antitumor effect by increasing the expression of endogenous miR-34a and decreasing the expression of drug resistance genes. The authors demonstrated that DTX/P-RUB micelles, not DTX or RUB, inhibited the proliferation of taxane resistant (TXR) prostate cancer cells and induced cell cycle arrest at G2/M phase. Moreover, PC3-TXR nude mice which treated with DTX/P-RUB micelles showed smaller tumor volume and lower tumor burden. The polymeric delivery system exhibited high antitumor activity via integrating miR-34a and DTX [99]. Ideal polymeric delivery vectors can be achieved by choosing desired materials. A polymer nanosystem vector namely ROSE which based on polyethylenimine and cyclodextrin was exploited to deliver miR-34a. The results showed that ROSE/miR-34a therapy inhibited tumor growth in mice bearing xenograft HCC tumors [100]. 7C1, a kind of nanoparticle polymeric vector, was used to deliver miR-34a systemically in a LAC model. In this model, the tumor progression was attenuation. And the anticancer effect became more prominent in condition of treatment with miR-34a and siRNA-Kras together [101].

### The application of miR-34a therapeutics on clinical

An effective delivery vector provides possibility for miR-34a to overcome numerous extracellular and intracellular obstacles, it is the guarantee for miR-34a to exert anti-tumor effect. miR-34a therapeutics get incredible success relied on various nanocarriers and numerous preclinical studies demonstrated the broad application prospect of miR-34a in oncotherapy, but researches are more than this. In April 2013, MRX34, a special amphoteric lipid nanoparticle filled with miR-34 mimics, as the first microRNA-associated therapeutic drug was tested in a clinical trial (NCT01829971). This trial recruited 155 participants, 7 cancer types in all, including primary liver cancer, several solid tumors and hematopoietic malignancies. Although some adverse immune response happened, this clinical trial provided a direction for MRX34 application on cancer therapy.

In recent years, many researches about MRX34 have been carried on various cancer and received something desired. Systemic delivery of MRX34 in mice bearing liver tumor xenografts resulted in about 1000-fold increased expression of miR-34a and the inhibition of tumor growth. Furthermore, MRX34 induced tumor regression in more than one third of mice [102]. Besides, A NSCLC mouse model (344SQ) which treated with MRX34 showed low expression of PDL1 in both gene and protein level. MRX34 treatment in 344SQ mouse model resulted in increased tumor-infltrating CD8+ cells and decreased tumor-infltrating PD1+ T-cells, macrophages and T-regulatory cells, and finally delayed tumor growth [103]. Moreover, NOV340, the encapsulated vehicle in the clinical trial, was also used to co-deliver miR-34a and *let-7b* to NSCLC mice which resistance to conventional anticancer therapy. As expected, the dual treated animals showed reduced tumor burden and prolonged survival [93]. To assess the safety, maximum tolerated dose (MTD) and clinical activity of MRX34, Beg et al [104] enrolled forty-seven patients with various cancers, including eleven cancer types. The authors found that these patients who were treated with MRX34 showed several adverse events including fever, fatigue etcetera. The MTD was 110 mg/m^2^ for non-HCC patients and 93 mg/m^2^ for HCC patients. Notably, MRX34 indeed exhibited antitumor activity in these patients with refractory solid tumors [104]. Even more noteworthy is the biodistribution of MRX34, it was found to be existed in various tissues including liver, bone marrow, spleen, mammary gland, lung etcetera [105]. The broad distribution of MRX34 allows the application in treating with numerous cancer types.

## Conclusion

The poor prognosis of cancer is largely ascribed to the metastasis of cancer cells. miR-34 family act as a negative regulatory factor of tumor associated-EMT and plays a considerable role in repressing tumorigenesis and retarding tumour progression. As an excellent tumor suppressor, miR-34a is considered for cancer therapy. A large number of studies about miR-34a therapeutics have been carried out, and verified its tumor-supressive role in cancer. However, some challenges are emerged along with the application of miR-34a therapeutics. One is the miRNA degradation mentioned above. RNase is abundant in serum and easily denatures miR-34a, resulting in that miR-34 cannot penetrate capillary endothelium and not to reach at target cells. Moreover, immunoreaction of miR-34a therapeutics is also deserved attention. In August 2016, MRX34 was tested in a clinical trial (NCT02862145) again, nevertheless it was withdrawn soon because of five immune related adverse events occurred. miR-34a therapeutics is depending on nanocarriers, the toxicity of nanoparticle is also worthy of discussion. In addition, some other unexpected side effects may come up, such as the accumulation of therapeutic miRNAs at healthy tissues because of the conjunction of serum proteins on the surface of nano-vectors, the breakdown of nanoparticles on the account of the shearing stress which from the flowing blood, the unsuccessful extravasation of nanocarriers to target cells by reason of the interstitial fluid pressure, and so on [106]. Even so, miR-34a is also a promising cancer therapeutic candidate. Besides, other members of miR-34 family have also been reported to inhibit proliferation of tumor cells. Notwithstanding few application of miR-34b/c in vivo, it is worthy to explore for oncotherapy.

## Abbreviations

AD: Alzheimer’s disease
AML: Acute myeloid leukemia
BC: Breast cancer
bHLH: Basic helix-loop-helix
BSCS: Breast cancer stem cells
ccRCC: Clear cell renal cell carcinoma
CHOL: Cholesterol
CLL: Chronic lymphocytic leukemia
CRC: Colorectal cancer
DODAP: 1,2-dioleyl-3-dimethylammonium propane
DSPC: Disteroylphosphatidylcholine
DTX: Docetaxel
EHCC: Extrahepatic cholangiocarcinoma
EMT: Epithelial-mesenchymal transition
EMT-TFs: Epithelial-mesenchymal transition transcription factors
EOC: Epithelial ovarian cancer
ESCC: Esophageal squamous cell carcinoma
GBC: Gallbladder cancer
GSH: Glutathione
HB: Hepatoblastoma
HCC: Hepatocellular carcinoma
HD: Huntington’s disease
HNSCC: Head and neck squamous cell carcinoma
hTERT: Human telomerase reverse transcriptase
IL-6R: Interleukin-6 receptor
LAC: Lung adenocarcinoma
LEF1: Lymphoid enhancer-binding factor-1
LSCC: Laryngeal squamous cell carcinoma
miR-34: microRNA 34
miRNAs/miRs: microRNAs
MM: Multiple myeloma
MMP9: Matrix metalloproteinase 9
mRNAs: Messenger RNAs
MTC: Medullary thyroid carcinoma
MTD: Maximum tolerated dose
NLE: Neutral lipid emulsion
NSCLC: Non-small cell lung cancer
OC: Ovarian cancer
OCSS: Oral squamous cell carcinoma
OMet: 2’-O-methylated
OSA: Osteosarcoma
PCa: Prostate cancer
PD: Parkinson’s disease
PDAC: Pancreatic ductal adenocarcinoma
PEG-2000: Poly ethylene glycol 2000
p-SMAD3: Phosphorylation of mothers against decapentaplegic 3
RUB: Rubone
SCC: Squamous cell carcinoma
SCID: Severe combined immunodeficien
SCLC: Small cell lung cancer
SGT: Salivary gland tumor
SNALPs: Synthesized stable nucleic acid lipid particles
STAT3: Signal transducer and activator of transcription 3
Tf: Transferrin
TGF-β: Transforming growth factor beta
TGF-βR1: Transforming growth factor beta receptor 1
TXR: Taxane resistant
UBC: Urothelial bladder cancer
UTRs: Untranslated regions
VISA: VP16-GAL4-WPRE integrated systemic amplifier
